# Oocyte CTR1 is not essential for cisplatin-induced oocyte death of primordial follicle

**DOI:** 10.17912/micropub.biology.000632

**Published:** 2022-09-01

**Authors:** Seok-Yeong Yu, Yi Luan, Amirhossein Abazarikia, Rosemary Dong, Jaekwon Lee, So-Youn Kim

**Affiliations:** 1 Olson Center for Women’s Health, Department of Obstetrics and Gynecology, College of Medicine, University of Nebraska Medical Center, Omaha, NE; 2 Department of Biochemistry, University of Nebraska, Lincoln, Nebraska, USA.

## Abstract

Accumulated evidence indicates that cisplatin, a platinum-based alkylating agent, causes preferential DNA damage to oocytes of primordial follicles (PFs) in the ovary, suggesting oocyte-favored accumulation of cisplatin. Copper transporter 1 (CTR1; *Slc31a1* ) is implicated in facilitating cisplatin uptake in cells. Here we found that oocytes of PFs had constitutively higher expression of CTR1 than other cell types in mouse ovary. However, oocyte-specific *Slc31a1* knockout was not sufficient to prevent cisplatin-induced depletion of PFs *in vitro* . Our data indicate that CTR1 would not be the only route for cisplatin to be transported inside the oocytes of PFs in the ovary.

**Figure 1. The distribution of CTR1 protein in the mouse ovary and effects of oocyte-specific Ctr1 knockout on ovarian reserve against cisplatin:**
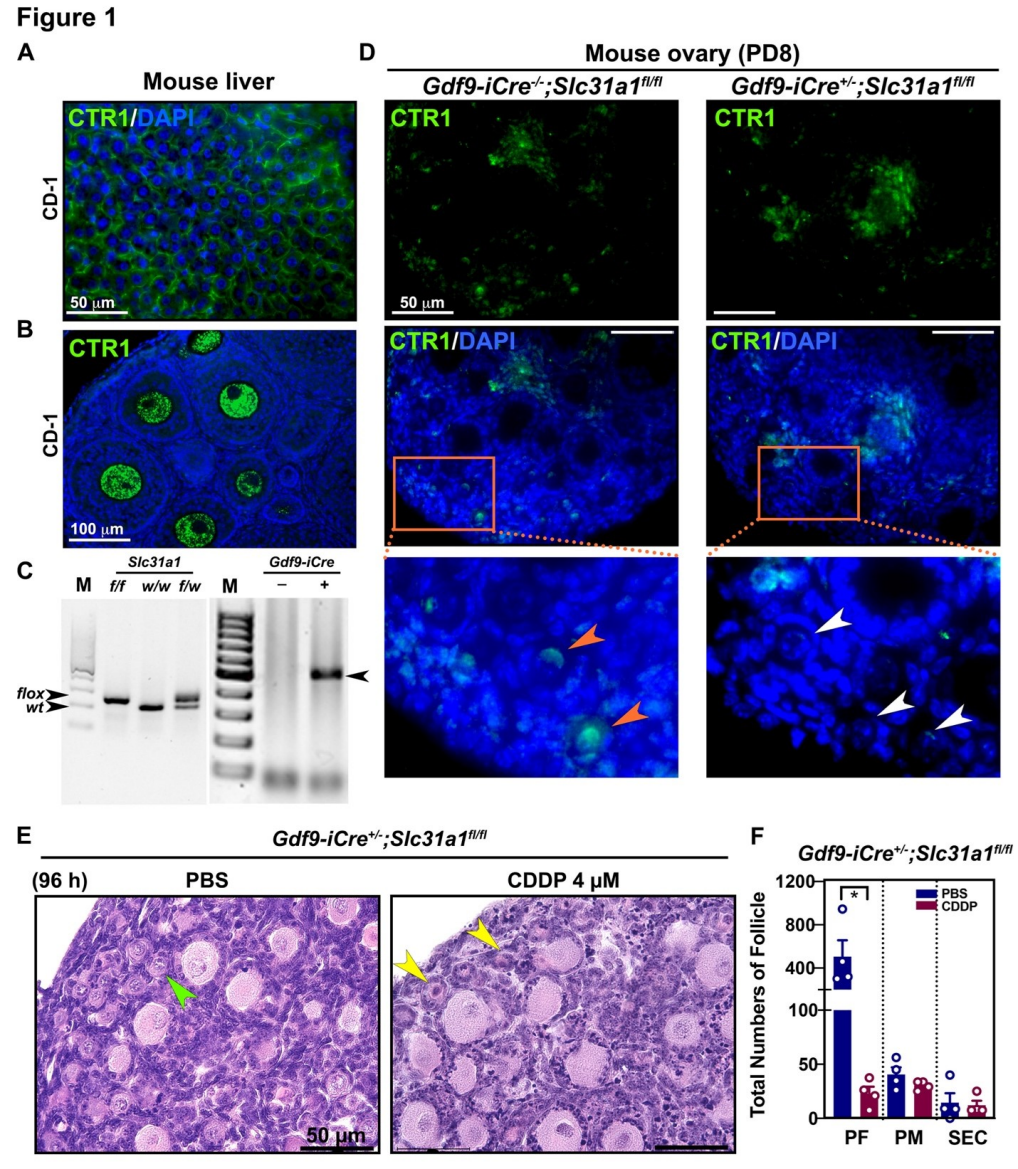
A and B. CTR1 immunofluorescence assay on the mouse liver and ovary sections. C. Representative genotyping image of homozygous or heterozygous for the floxed *Slc31a1* allele and *Gdf9-iCre* transgene. D. CTR1 immunofluorescence assay on ovary sections from wild-type or oocyte-specific *Slc31a1* knockout mice. Zoomed pictures are located below each panel. The orange arrows for PFs from wild-type ovary indicate the expression of CTR1 in oocytes of PFs. The white arrows for oocytes of PFs exhibit the absence of CTR1 expression from oocyte-specific *Slc31a1* knockout ovary. E. Representative image of H&E staining of ovary sections from oocyte-specific *Slc31a1* knockout PD8 mice *ex vivo* cultured in the presence or absence of cisplatin ( *cis* -diamminedichloroplatinum(II), CDDP) for 96 hours. Green arrow, surviving PF; Yellow arrows, dead PFs. F. Total numbers of follicles (n=4). Scale bar for A, D, and E, 50 μm; Scale bar for B, 100 μm. *, *p* < 0.05.

## Description

Chemotherapeutic drugs are primarily intended to treat metastatic and/or unresectable cancers by inducing systemic cancer cell apoptosis. Alkylating chemotherapy induce apoptosis of proliferative cells by binding to DNA and causing persistent DNA adducts. Cisplatin [Pt(NH _3_ ) _2_ Cl _2_ ; CDDP] has been the first-line chemotherapy for various cancer types and has increased survival rates in cancer patients (Brown et al., 2019). Prescribed doses of CDDP range 30 to 100 mg/m ^2^ weekly giving the peak concentration in circulation around 10 to 50 μM, although the treatment scheme is susceptible to changes by cancer type and status (van Hennik et al., 1987). However, CDDP accompanies off-target effects such as nephrotoxicity, cardiotoxicity, and infertility, all of which compromise the quality of life of patients (Ahmad et al., 2016). Nonetheless, CDDP is still widely used to treat pediatric and young adults diagnosed with multiple cancer types including germ cell tumors, hepatoblastoma, and osteosarcoma (Moke et al., 2021).

Primordial follicle (PF) consisting of ovarian reserve has an oocyte surrounded by a single layer of flattened granulosa cells. A finite number of PFs is set around birth in women and steeply decreases by puberty (Liew et al., 2017). The number and quality of PFs in the pool determines a woman’s reproductive life. Therefore, CDDP-induced ovarian follicle loss in women of reproductive age is the most critical health issue as oocytes in the ovary cannot be replenished. Potential mechanisms by which CDDP causes premature ovarian insufficiency (POI) in women have been addressed over the recent decade. It is demonstrated that CDDP is preferentially accumulated in the oocytes of PFs as indicated by immunofluorescence assay for platinum (Pt) in mouse ovaries treated with CDDP (Kim et al., 2013). In addition, CDDP is shown to dramatically decrease the number of PFs in the ovaries (Eldani et al., 2020; Kim et al., 2013; Kim et al., 2019; Nguyen et al., 2019). However, knockout of proapoptotic gene *Puma* prevented CDDP-induced loss of PFs in the mouse ovaries (Nguyen et al., 2018). Consistently, our previous studies demonstrated that oocyte-specific knockout of *Trp63* , which is the upstream regulator of *Puma* , rescued CDDP-induced depletion of PFs (Kim et al., 2019). These observations suggest that CDDP mediates PF depletion largely through the apoptotic pathway in oocytes of PFs, which would underlie the development of POI in women. Thus, it raised the question of how CDDP preferentially targets oocytes of PFs among other cell types in the ovaries.

There are two major ways that convey CDDP across cell membrane: active or passive uptake. Active uptake utilizes transporters such as the copper transporter 1 (CTR1), the organic cation transporter 2 (OCT2), and the multidrug extrusion transporter 1 (MATE1) (Ciarimboli, 2012). However, the varying tissue distribution of the transporters indicate that the primary transporter for CDDP would differ by tissues. For example, CTR1 is ubiquitously expressed in the intestines, brain, kidney, liver, eye, testes, and ovary in mice (Kuo et al., 2001; Lee et al., 2000). OCT2 is mainly expressed in the liver and kidney (Nakata et al., 2013). On the other hand, passive uptake indicates the direct diffusion of uncharged, lipophilic molecules through the bilayer of the cell membrane in a concentration-dependent manner. Since CDDP is lipophilic and has no net charge, it is assumed that passive diffusion through the cell membrane in part mediates intracellular accumulation of CDDP (Eljack et al., 2014).

CTR1 that is encoded by the *SLC31A1* gene is ubiquitously expressed in various organs including the ovary and plays a facilitating role in CDDP uptake in adult patients (Ishida et al., 2002; Urien and Lokiec, 2004). The high susceptibility of oocytes of PFs to CDDP-induced apoptosis might reflect cell-specific distribution of CTR1 expression in the ovary. Therefore, we characterized the distribution of CTR1 protein in the mouse ovary and examined whether *solute carrier family 31 member 1* ( *Slc31a1)* knockout rescued oocytes of PFs from CDDP-induced depletion *ex vivo* .

Antibody specificity for CTR1 was confirmed using mouse liver and ovary sections at postnatal day 28 (PD28) (Figs. 1A and B). Then, ovaries were harvested from female pups at PD8, and polymerase chain reaction (PCR) was performed with gDNA from their tails for genotyping. The floxed *Slc31a1* allele and *Gdf9-iCre* transgene were confirmed by PCR band sizes (Fig. 1C): *Gdf9-iCre ^-/-^ ;Slc31a1 ^fl/fl^* and *Gdf9-iCre ^+/-^ ;Slc31a1 ^fl/fl^* are considered wild-type and oocyte-specific *Slc31a1* knockout, respectively. Immunofluorescence assay for CTR1 revealed that oocytes in PFs had relatively higher expression levels of CTR1 than other cell types in the wild-type ovary at PD8 (Fig. 1D, left panel). Weak signals of CTR1 were also detected in the oocytes and granulosa cells in growing follicles and stromal cells of the ovary. However, CTR1 was not detected in the oocytes but only found in the granulosa and stromal cells of the *Slc31a1* knockout ovary (Fig. 1D, right panel), confirming oocyte-specific knockout of *Slc31a1* in the ovary at PD8. Next, we cultured ovaries *ex vivo* from the knockout mice in the presence or absence of CDDP at 4 μM. The dose of cisplatin that we chose stays within the plasma concentrations found in the patients who received a single does of cisplatin (30 mg/m ^2^ ). Thus, 4 μM of cisplatin would be ideal to test the importance of CTR1 in cisplatin transport in oocytes. Nonetheless, cisplatin would be largely transported through passive diffusion when concentration is too high. Histological comparison indicates that CDDP caused the death of oocytes of PFs in the oocyte-specific *Slc31a1* knockout (Fig. 1E). Follicle counting data demonstrate that CDDP significantly reduced the number of PFs in the oocyte-specific *Slc31a1* knockout compared to non-treated ovaries (Fig. 1F). Therefore, these data indicate that oocyte-specific *Slc31a1* knockout was not enough to prevent CDDP-induced death of oocytes in primordial follicles in the current experimental setting. The null findings would be accounted for by 1) CTR1 might not be the only route for CDDP to enter oocytes in the primordial follicles or 2) the concentration of 4 μM would be so high that CDDP is rather transported through passive diffusion. These limitations should be taken into consideration for interpreting the current data.

## Methods

**Animals**

*Gdf9-iCre* male mice were purchased from Jackson Laboratories, generated and characterized previously (Kim et al., 2009). *SLC31A1 ^fl/fl^* strain on the C57BL/6 background was obtained from Dr. Jaekwon Lee. All procedures involving the use of mice were approved by the Institutional Animal Care and Use Committee (IACUC) at the University of Nebraska Medical Center (UNMC). Animals were provided with food and water *ad libitum* and maintained in the Comparative Medicine facilities of UNMC.

**Genotyping**

For floxed *Slc31a1* and *Gdf9-iCre* genotyping, we used the following sets of primers; floxed *Slc31a1* (Primer 1: 5’-AAT GTC CTG GTG CGT CTG AAA-3’ & Primer 2: 5’-GCA GTA GAT AAA AGC CAA GGC-3’) and *Gdf9-iCre* (Primer 1: 5’-GAT ATC AAG CTT GTC CAC CAT GGT G-3’ & Primer 2: 5’-CAA CTC TAG ACT CAG TTT CAG TCC CCA TC-3’).

**Whole-ovary organ culture *in vitro***

Whole-ovary organ culture was performed as previously described with some modification (Kim et al., 2013). Briefly, isolated ovaries from mice at PD8 were cultured on 0.4 μm Millicell Cell Culture Inserts (MilliporeSigma, PICM01250) in a 24-well plate in the Minimum Essential Medium α (Gibco, 32571036) supplemented with 1 mg/ml bovine serum and 5 μg/ml insulin-transferrin-selenium (Gibco, 41400045). The media were changed after 48 hours, and the ovaries were fixed for staining after another 48 hours.

**Immunofluorescence assay**

The ovaries were fixed, embedded in paraffin, and sectioned at 5 μm. For immunofluorescence assay, sectioned tissues were blocked in 2% goat-serum blocking solution for an hour and incubated with CTR1 antibody (1:50, Cell Signaling Technology, #13086S) overnight. In the following day, the sections were incubated with anti-rabbit Alexa Fluor488 (1:200, Invitrogen, A-11008) and stained with DAPI. All the images were taken with the EVOS M7000 Imaging System (Invitrogen, AMF700).

**Hematoxylin/eosin (H&E) staining and Follicle counting**

Tissues were serially sectioned at 5 μm thickness. Sectioned ovaries were stained with H&E and subject for histological examination and follicle counting as demonstrated previously (Kim et al., 2015).

**Statistical analysis**

Graphs were generated by Prism 9.1.1 software (GraphPad Software Version 9, Inc.), and data were presented as mean ± S.E.M. Unpaired two-tailed Student's t-test was used to compare the means between two datasets. *P* values of less than 0.05 were considered statistically significant. n.s. represents not-significant and * represents *p* < 0.05.

## Reagents

**Table:** 

**Strain**	**Genotype**	**Stock#**	**Available from**
* Ctr1 ^flox^ / ^flox^*	C57BL/6NJ-Slc31a1 ^tm2djt^ /J		Dr. Jaekwon Lee, UNL
*Gdf-9-iCre*	C57BL/6NJ- Tg(Gdf9-icre)5092Coo	011062	Jackson Laboratory

## References

[R1] Ahmad SS, Reinius MA, Hatcher HM, Ajithkumar TV (2016). Anticancer chemotherapy in teenagers and young adults: managing long term side effects.. BMJ.

[R2] Brown A, Kumar S, Tchounwou PB (2019). Cisplatin-Based Chemotherapy of Human Cancers.. J Cancer Sci Ther.

[R3] Ciarimboli G (2012). Membrane transporters as mediators of Cisplatin effects and side effects.. Scientifica (Cairo).

[R4] Eldani M, Luan Y, Xu PC, Bargar T, Kim SY (2020). Continuous treatment with cisplatin induces the oocyte death of primordial follicles without activation.. FASEB J.

[R5] Eljack ND, Ma HY, Drucker J, Shen C, Hambley TW, New EJ, Friedrich T, Clarke RJ (2014). Mechanisms of cell uptake and toxicity of the anticancer drug cisplatin.. Metallomics.

[R6] Ishida S, Lee J, Thiele DJ, Herskowitz I (2002). Uptake of the anticancer drug cisplatin mediated by the copper transporter Ctr1 in yeast and mammals.. Proc Natl Acad Sci U S A.

[R7] Kim H, Son HY, Bailey SM, Lee J (2008). Deletion of hepatic Ctr1 reveals its function in copper acquisition and compensatory mechanisms for copper homeostasis.. Am J Physiol Gastrointest Liver Physiol.

[R8] Kim SY; Cordeiro MH; Serna VA; Ebbert K; Butler LM; Sinha S; et al.; Kurita T. 2013. Rescue of platinum-damaged oocytes from programmed cell death through inactivation of the p53 family signaling network. Cell Death Differ 20: 987-97. 10.1038/cdd.2013.31 PMC3705595 23598363

[R9] Belda Júnior W, Cucé LC, Dias MC, Lacaz Cda S. 1989. [Black grain mycetoma caused by Madurella grisea]. Rev Inst Med Trop Sao Paulo 31: 195-9. 10.1590/s0036-46651989000300010 2559470

[R10] Kim SY, Nair DM, Romero M, Serna VA, Koleske AJ, Woodruff TK, Kurita T (2018). Transient inhibition of p53 homologs protects ovarian function from two distinct apoptotic pathways triggered by anticancer therapies.. Cell Death Differ.

[R11] Kuo YM, Zhou B, Cosco D, Gitschier J (2001). The copper transporter CTR1 provides an essential function in mammalian embryonic development.. Proc Natl Acad Sci U S A.

[R12] Lee J, Prohaska JR, Dagenais SL, Glover TW, Thiele DJ (2000). Isolation of a murine copper transporter gene, tissue specific expression and functional complementation of a yeast copper transport mutant.. Gene.

[R13] Liew SH, Nguyen QN, Strasser A, Findlay JK, Hutt KJ (2017). The ovarian reserve is depleted during puberty in a hormonally driven process dependent on the pro-apoptotic protein BMF.. Cell Death Dis.

[R14] Moke DJ, Luo C, Millstein J, Knight KR, Rassekh SR, Brooks B, Ross CJD, Wright M, Mena V, Rushing T, Esbenshade AJ, Carleton BC, Orgel E (2021). Prevalence and risk factors for cisplatin-induced hearing loss in children, adolescents, and young adults: a multi-institutional North American cohort study.. Lancet Child Adolesc Health.

[R15] Nakata T, Matsui T, Kobayashi K, Kobayashi Y, Anzai N (2013). Organic cation transporter 2 (SLC22A2), a low-affinity and high-capacity choline transporter, is preferentially enriched on synaptic vesicles in cholinergic neurons.. Neuroscience.

[R16] Nichols WW, Milne LM (1986). Derepressed beta-lactamase synthesis in strains of Pseudomonas aeruginosa isolated from patients with cystic fibrosis.. J Antimicrob Chemother.

[R17] Nguyen QN, Zerafa N, Liew SH, Morgan FH, Strasser A, Scott CL, Findlay JK, Hickey M, Hutt KJ (2018). Loss of PUMA protects the ovarian reserve during DNA-damaging chemotherapy and preserves fertility.. Cell Death Dis.

[R18] Urien S, Lokiec F (2004). Population pharmacokinetics of total and unbound plasma cisplatin in adult patients.. Br J Clin Pharmacol.

[R19] van Hennik MB, van der Vijgh WJ, Klein I, Elferink F, Vermorken JB, Winograd B, Pinedo HM (1987). Comparative pharmacokinetics of cisplatin and three analogues in mice and humans.. Cancer Res.

